# Simultaneous Estimation of Twenty Eight Phenolic Compounds by a Novel and Expeditious Method Developed on Quaternary Ultra-Performance Liquid Chromatography System with a Photodiode Array Detector

**DOI:** 10.3390/biom10010006

**Published:** 2019-12-18

**Authors:** Shiwani Mandhania, Ajay Pal, Vinod Saharan

**Affiliations:** 1 Cotton Biochemistry Laboratory, Cotton Section, Department of Genetics and Plant Breeding, CCS Haryana Agricultural University, Hisar, Haryana 125 004, India; 2 Department of Biochemistry, College of Basic Sciences and Humanities, CCS Haryana Agricultural University, Hisar, Haryana 125 004, India; 3 Nano Research Facility Lab, Department of Molecular Biology and Biotechnology, Maharana Pratap University of Agriculture and Technology, Udaipur, Rajasthan 313 001, India

**Keywords:** UPLC, PDA, Phenolics, Flavonoids, Cotton, Root rot

## Abstract

Plant secondary metabolites including phenolics and flavonoidsare synthesized through phenylpropanoid and phenylpropanoid–acetate pathways and significantly contribute against adverse effect of abiotic and biotic stresses. Herein, we present the development and execution of a novel and expeditious ultra-performance liquid chromatographic-photodiode array (UPLC–PDA) method for qualitative and quantitative analysis of 28 phenolic compounds comprising of flavonoids, phenolic acids, aldehydes and alcohols. The method is able to separate phenolic compounds in just 17 min with the separation of isobaric species such as 3,4 dihydroxybenzoic acid and 3,5 dihydroxy benzoic acid; quercetin and taxifolin. Linear curves concentrations ranged from 6–18 µg/mL (3,5 dihydroxy benzoic acid), 4–12 µg/mL (catechin and salicylic acid) and 2–6 µg/mL for rest of the compounds and correlation coefficients were >0.994. The limit of detection (LOD) varied from 0.04–0.45 µg/mL. Cotton root samples were used to assess the method in terms of recovery efficiency (85–120%), precision (0.12–4.09%) and intermediate precision (0.32–4.0%).Phenolics and flavonoidsin root samples of healthy and diseased plants as well as leaf samples of healthy plants were successfully quantified using this novel method without an expensive Mass Spectrometer.

## 1. Introduction

**Figure 1:**
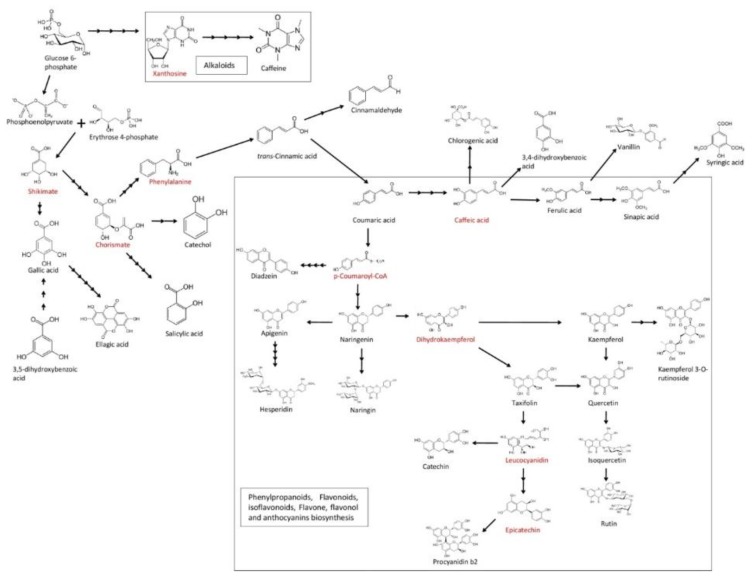
Schematic diagram of the phenylpropanoid biosynthetic pathway.

Cotton, also known as “white gold” is grown as a fiber and food crop all over the world. Amongst the major cultivated species of cotton, *Gossypium hirsutum* L. significantly contributes to total lint cotton production [1,2]. India ranks first in terms of area of cultivation but lags behind in productivity, which is severely affected by various abiotic and biotic stresses [3]. Cotton plants are attacked by 1326 species of insects and dozens of pathogens [4]. Among all, cotton leaf curl virus and *R. solani* severely affect the productivity and accounts for up to 40% reduction in yield [5,6]. The infection and infestation of plants lead to activation of protein inhibitors and induction of defense mechanisms including the release of secondary metabolites like phenolic compounds [7,8]. Phenylalanine and some phenolic compounds are derived from phosphoenol phosphate and erythrose-4-phosphate through the shikimate pathway. The former is converted into cinnamic acid which acts as a precursor for biosynthesis of phenolic compounds. Cinnamic acid via coumaroyl-CoA is routed through the phenylpropanoid pathway for synthesis of additional phenolics and lignin polymerization. Alternatively, it is routed through a phenylpropanoid–acetate pathway for the biosynthesis of flavonoids and isoflavonoids (Figure 1). The diversity of phenolics with acid, aldehyde or alcohol groups and their acylation and glycosylation make the process of separation and quantification difficult in complex plant matrices. Currently, several methodologies are in use for the analysis of phenolic compounds in plant systems. Amongst them, spectrophotometric-based Folin–Denis and differential pH assays are preferably used for quantification of total phenolics and anthocyanins, respectively [8]. However, these methods lack the specificity as well as selectivity which lead to over or underestimation of phenolic content [9]. So, there is an urgentnecessity to quantify the differential phenolics using a reliable, rapid and sensitive analytical technique to provide raw screened breeding materials to plant pathologists and breeders to boost pathway discovery as well as metabolic engineering [10,11]. Amongst the various analytical techniques being currently practiced for separation and quantification of phenolics, a high-performance liquid chromatography–photodiode array detector (HPLC–PDA) using a reverse-phase C18 column is commonly applied [12,13,14,15]. Some free and bound phenolic acids as well as flavonoids, for example, have been simultaneously quantified in food and plant samples using this technique [16,17]. The chemical diversity and complexity of these compounds have been analyzed through an untargeted approach using high-resolution mass spectrometry [18,19,20,21], whereas triple quadruple has been used to specifically quantify the targeted compounds. However, only phenolics or flavonoids have been considered at a time in most of the studies [22,23]. Very few studies have focused on the simultaneous estimation of all types of phenolic compounds including acid, aldehyde and flavonoids [11,24,25]. Moreover, none of the studies have accomplished the simultaneous separation and quantification of phenolics, phenolic acid, derivatives of hydroxycinnamic acid and flavonoids which are crucial for lignin biosynthesis.

The present work illustrates the development of a method to separate and quantify 28 phenolic compounds of different chemistry using ultra-performance liquid chromatography system equipped with a photodiode array detector (UPLC–PDA). The study achieved a secondary metabolites chromatographic resolution including isobaric species in less than 19 min. The developed method is fully validated for simultaneous identification of 28 phenolic compounds to characterize leaf and root samples of cotton.

## 2. Materials and Methods

### 2.1. Chemicals

Acetonitrile and formic acid of HPLC grade were supplied by Merck-Sigma (Germany). Ultrapure water was obtained from Millipore Q 8 System (Millipore, MA, USA).

### 2.2. Standards

Trans-cinnamic acid; 3,5 dihydroxybenzoicacid (3,5 DHBA); chlorogenic acid; catechin; 2-methoxycinnmaldehyde (2–MC); ellagic acid; (±) -naringenin; naringin; procyanidin B2; trans-sinapic acid; taxifolin were procured from Sigma Aldrich (St Louis, MO, USA). Rutin hydrate; syringic acid; kaempferol; isoquercetin; kaempferol-3-O-β rutinoside (K-3-O-βR); daidzein were obtained from Sigma (St Louis, MO, USA). Gallic acid; caffeine; vanillin; salicyclic acid; 3,4 dihyroxybenzoic acid (3,4 DHBA); trans-ferulic acid; trans–*p*-coumaric acid were procured from Fluka (St Louis, MO, USA). Catechol; hesperdin; apigenin; quercetin dihydrate were purchased from Alfa Acer, Thermo Fisher Scientific (USA). Stock solutions (2000 ppm) of individual compound were prepared in methanol. Two separate intermediate solutions of all the standards were prepared. One was used for the estimation of LOQ (limit of quantification) and LOD (limit of detection) while the other for recovery and linearity by diluting in methanol as depicted in Table 1. The working solution was further prepared by diluting in mobile phase A and B in the ratio 80:20.

### 2.3. Samples

Root and leaf samples of cotton plant were taken from the healthy and sick plot area (field area maintained for growth and maintenance of *Rhizoctonia* culture for screening of cotton root rot tolerant genotypes) of cotton research farm area, CCS Haryana Agricultural University, Hisar, in *Kharif* season 2018. Root samples collected from healthy and sick plot area served as sample D (healthy) and C (diseased), respectively while leaf sample from healthy plant served as sample L. Phenolic compounds were extracted from root (0.2 g) and leaf (2.0 g) samples following the method of Adomand Liu [26]. The extracted samples were further cleaned using Sep-Pak C18 and Oasis HLB 6CC cartridges (Waters MA, USA) as described earlier [27] with minor modifications. The samples were finally dissolved in methanol and filtered through nylon 0.22-micron syringe filters.

### 2.4. Instrumentation

Phenolic compounds were analyzed using quaternary ultra-performance liquid chromatography (UPLC) equipped with a photodiode array detector (PDA) (H-Class Acquity, Waters, Milford, MA, USA). The BEH HSS C 18 column having 1.7 μm particle size and 2.1 mm inlet diameter was used to resolve 28 phenolics. To achieve the resolution, different gradient systems with varied concentrations of additive (formic acid) was examined. Acetonitrile (solvent B) and 0.01% formic acid in water (solvent A) with a flow rate of 600 µL/minute proved best for resolution of phenolic compounds and isomers of phenolics and flavonoids under study (data not shown) at 35 °C column temperature. The optimized gradient elution began with 95% (solvent A) and adjusted to 85% at 8.30 min; 80% (solvent A) and 20% (solvent B) at 10.60 min; 70% (solvent A) and 30% (solvent B) at 12.90 min; 50% of both solvents (A and B) at 13.90 min and continued up to 14.60 min. The column was conditioned with an initial injection condition of 95% (A) and 5% (B) from 14.65 to 17.00 min and next injection was delayed by 2.0 min. The absorbance spectrum of standards was obtained in the range 190–400 nm using a PDA detector to find wavelength maxima (λ_max_). The λ_max_ of individual 28 phenolic compounds in mixed solution is given in Table 1. Based on peak intensity measured, a wavelength of 278 nm was chosen for detection of all the phenolic compounds.

### 2.5. Analytical Method Validation

The characteristics of method performance were established with assays of blanks, standards, selectivity and system suitability, linearity, recovery, precision, intermediate precision, LOD and LOQ at 5% significance level.

#### 2.5.1. Selectivity and System Suitability 

Peak purities of 28 standards and a root sample of cotton spiked with these standards were determined by analyzing the PDA spectra at different peak points. System suitability was gauged by examining the consequences from USP criteria obtained at 100% level of concentrations with six injections.

#### 2.5.2. Linearity 

Five variable concentrations (50, 75, 100, 125 and 150%) including one base concentration (100%) of 28 standards were prepared from mixed solution. The base concentration was 8 µg/mL for catechin and salicylic acid; 12 µg/mL for 3,5 dihydroxy benzoic acid and 4 µg/mL for rest of the phenolic compounds. Calibration curves were made from these concentrations, analyzed in duplicate over UPLC–PDA and regression analysis was computed through built-in Empower 3 software.

#### 2.5.3. Precision and Accuracy

Analysis of duplicate injections of two different samples in two replications was carried out on same and different days to check the intra and inter-day precision (intermediate precision). Relative standard deviation (RSD) of the values expressed the precision of method.

For estimation of recovery, unfiltered sample was spiked with three concentrations (75, 100, 125%) of standards. The developed method was applied to analyze two independent samples at each concentration and percent recovery was expressed as-

[Calculated concentration/Theoretical concentration] × 100(1)

#### 2.5.4. Limit of Quantification (LOQ) and Limit of Detection (LOD) 

Serial dilutions of standards were prepared by diluting in mobile phase A and B in the ratio 80:20 and used for estimation of LOD. The signal (s) to noise (n) ratios of 3 and 10 were used to express LOD and LOQ, respectively.

## 3. Results and Discussion

### 3.1. UPLC Method Development

**Figure 2:**
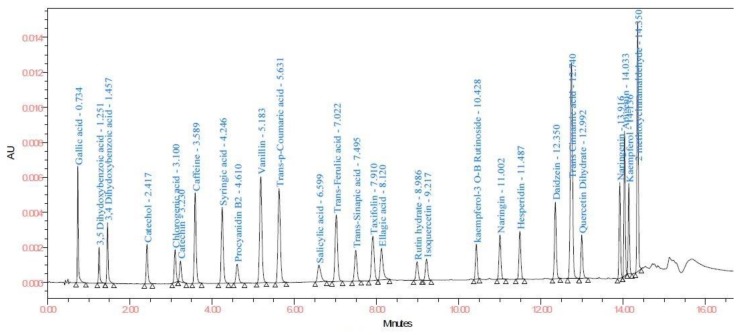
Chromatogram of standard phenolics at 278 nm.

HPLC methods used in cotton plant and food [12,15] were adopted for the maturation of present analytical method. The optimized UPLC mobile phase consists of (A) 0.01% formic acid in Milli Q water (pH = 3.34): (B) ACN system on a Waters column with an optimal gradient of 17 min. A total of 28 standards were identified to quantify the phenolic compounds in samples (Figure 2).

### 3.2. Analytical Method Validation

#### 3.2.1. Selectivity and System Suitability

Identification and quantification of 28 peaks from cotton root extract spiked with standard phenolics and related reference standards were reviewed (Supplementary Material A 1–3) and results show that other compounds did not co-elute (Table 2 and Table 3). 

Elution of *p*-coumaric acid, ferulic acid and sinapic acid with major phenolic acids usually requires 40–50 min by HPLC [28]. Mattila and Kumpulainen [16], however, reduced time to 27 min but ferulic and sinapic acids could not be resolved. But, in the present method system suitability indicates consistent chromatographic conditions like USP tailing < 1.5; USP resolution >1.5; selectivity >1; number of theoretical plates >7000, according to the US FDA (FDA, 1994) within 17 min.

#### 3.2.2. Linearity, LOQ and LOD

Linear curves regression formula and correlation coefficients of standards are presented in Table 3. Results indicate that linearity of all 28 standards varies from 0.994 to 0.998. Fracassetti et al. [29] prepared calibration curves of catechin and caffeic acid in the ranges from 0.5 to 80 μg/mL and 0.5 to 50 μg/mL, respectively for establishment of a UPLC method. In the present method, linear model was computed from the calibration curve (area *versus* amount, which fulfils the requirement of homoscedasticity (Supplementary Material B 1–28). 

The LOD was 0.04–0.450 µg/ml on the foundation of s/n ratio of 3 and the LOQ was 3.3 times of LOD (Table 3, Supplementary Material C 1-2). Our study corroborates with the findings of Dias et al. [30], Escarpa and González [31]. 

#### 3.2.3. Accuracy and Precision

The results presented in Table 4 show that recovery ranges from 85 to 112% which falls within the generally accepted range of 85 to 120%. Moreover, for recovery studies in quantitative analysis, there are no set official criteria and rely on analyte concentration as per AOAC guidelines [32]. The recovery of analyte at 75, 100 and 125% ranged from 85 to 109%, 85 to 105%, 87 to 103% and 85 to 112%, 87 to 105%, 85 to 99% in sample C and sample D, respectively. 

The repeatability RSD for precision varied from 0.12 to 2.96% and 0.62 to 4.09%, whereas, intermediate precision varied from 0.32 to 2.05% and 1.04 to 4.0% in sample C and D, respectively as depicted in Table 5. The better precision of the developed UPLC method as indicated by low values of RSD could be considered of high accuracy.

#### 3.2.4. Application of Method

**Figure 3:**
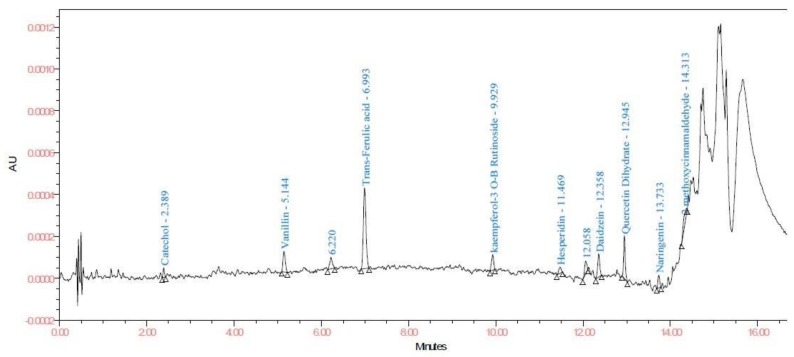
Chromatogram of diseased cotton root sample (sample C) at 278 nm.

**Figure 4:**
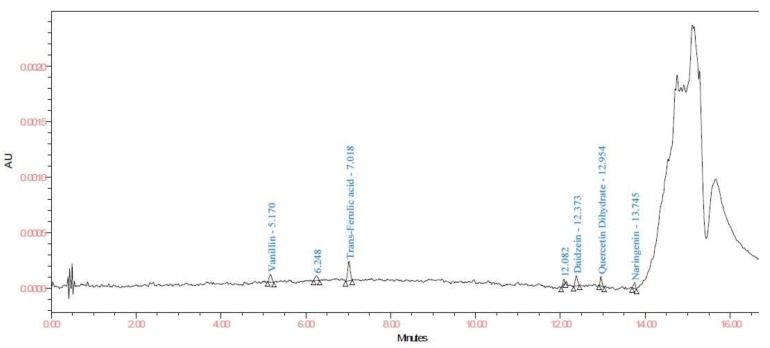
Chromatogram of cotton root sample (sample D) at 278 nm.

**Figure 5:**
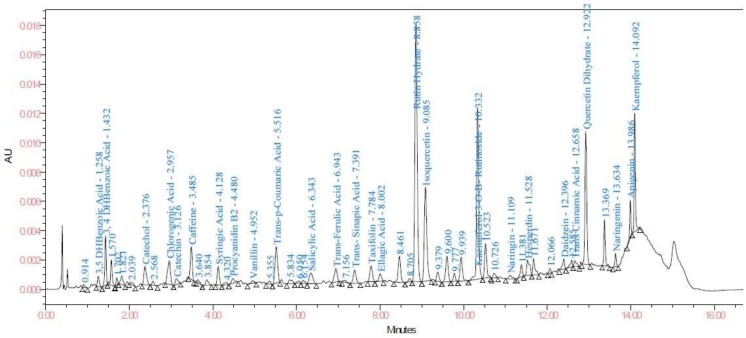
Chromatogram of healthy cotton leaf sample at 278 nm.

To test the feasibility of developed method, leaves of healthy cotton plant and roots of diseased (root rot) (sample C) and healthy (sample D) plants were analyzed for phenolic compounds. Catechol and caffeine were exclusively found in the diseased root sample. The diseased plant possessed significantly higher content of all quantified phenolic compounds except 2- methoxycinnamaldehyde which is higher in healthy root sample (Figure 3 and Figure 4). The highest concentration of rutin hydrate (22.05 µg/g) followed by isoquercetin (7.05 µg/g) was found in healthy leaf sample (Figure 5). Results of the study emphasizes that the developed procedure allows excellent resolution and sensitivity which further enable the estimation of very small amounts of phenolics in the sample. 

## 4. Conclusions

The present study describes the development of a model for simultaneous resolution and quantification of 28 phenolic compounds including isobaric species (3, 4 and 3, 5 dihydroxybenzoic acid; taxifolin and quercetin) with high accuracy and sensitivity using a UPLC–PDA system. The developed method was successfully applied in cotton leaves and roots to quantify the levels of various phenolic compounds. Findings of the study provide an option for swift and efficient quantification of plant phenolics at low cost. 

## Figures and Tables

**Table 1:** Concentrations of intermediate solutions and λ_max_ of standards.

Sr. No.	Compound	LOD and LOQ (μg/mL)	Linearity and Recovery (μg/mL)	λ_max_
1	Gallic acid	8.0	40.0	214.7
2	3,5DHBA	40.0	120.0	209.7
3	3,4DHBA	15.0	40.0	209.7
4	Catechol	20.0	40.0	209.7
5	Chlorogenic acid	20.0	40.0	324.9
6	Catechin	45.0	80.0	209.7
7	Caffeine	8.0	40.0	209.7
8	Syringic acid	10.0	40.0	216.8
9	ProcyanidinB2	30.0	40.0	209.7
10	Vanillin	20.0	40.0	229.7
11	Trans-*p*-coumaric acid	8.0	40.0	309.3
12	Salicylic acid	40.0	80.0	209.7
13	Trans-ferulic acid	15.0	40.0	321.3
14	Trans-sinapicacid	40.0	40.0	322,5
15	Taxifolin	8.0	40.0	209.7
16	Ellagic acid	40.0	40.0	253.4
17	Rutinhydrate	40.0	40.0	209.7
18	Isoquercetin	30.0	40.0	209.7
19	K-3-O-βR	30.0	40.0	209.7
20	Naringin	15.0	40.0	213.2
21	Hesperidin	15.0	40.0	209.7
22	Daidzein	15.0	40.0	248.7
23	Trans-cinnamic acid	8.0	40.0	277.1
24	Quercetin dihydrate	40.0	40.0	209.7
25	Naringenin	15.0	40.0	212.0
26	Apigenin	15.0	40.0	209.7
27	Kaempferol	20.0	40.0	209.7
28	2-MC	4.0	40.0	286.7

**Table 2:** Peak purities of standards, spiked sample C and D for assessment of system suitability of method.

Sr. No.	Compound	Peak Purity of Standard				Peak purity of Spiked Sample C				Peak Purity of Spiked Sample D			
		RT	Area	Purity Angle	Purity Threshold	RT	Area	Purity Angle	Purity Threshold	RT	Area	Purity Angle	Purity Threshold
1	Gallic acid	0.739	11215	0.719	2.660	0.739	10690	0.855	2.754	0.738	10905	0.758	2.798
2	3,5 DHBA	1.256	4009	0.230	1.966	1.258	4047	0.189	1.983	1.258	4119	0.249	2.058
3	3,4 DHBA	1.460	7211	0.436	2.288	1.463	7402	0.499	2.557	1.462	7332	0.454	2.525
4	Catechol	2.421	6245	2.004	4.969	2.412	6520	3.355	6.878	2.413	6597	4.331	6.885
5	Chlorogenic acid	3.100	5562	0.866	2.316	3.072	5758	1.054	2.586	3.079	5862	0.772	2.501
6	Catechin	3.232	3938	8.175	7.158	3.204	4306	9.411	9.754	3.211	4083	6.609	8.747
7	Caffeine	3.589	15622	0.648	2.712	3.552	16310	2.094	3.415	3.558	16204	1.370	3.516
8	Syringic acid	4.246	14732	0.635	2.705	4.208	15648	0.836	3.268	4.215	15669	0.761	3.246
9	Procyanidin B2	4.609	4456	2.879	6.805	4.567	4818	4.279	10.237	4.574	4650	4.204	10.033
10	Vanillin	5.186	23682	0.315	2.103	5.151	25149	0.370	2.301	5.158	25133	0.361	2.270
11	Trans-*p*-coumaric acid	5.634	21509	0.263	1.957	5.601	22689	0.285	2.049	5.609	22487	0.234	2.026
12	Salicylic acid	6.605	5213	0.905	3.261	6.575	5581	0.950	3.454	6.582	5444	0.901	3.465
13	Trans-ferulic acid	7.028	16178	0.258	1.991	6.996	18347	0.213	1.984	7.005	17770	0.257	2.063
14	Trans-sinapicacid	7.501	7192	0.276	2.013	7.468	7315	0.291	2.127	7.477	7289	0.278	2.125
15	Taxifolin	7.918	11366	0.727	2.826	7.884	11727	0.880	3.072	7.894	11904	0.776	3.183
16	Ellagic acid	8.130	8676	2.871	2.903	8.095	8598	3.021	3.438	8.106	8621	3.082	3.410
17	Rutin hydrate	8.997	3836	0.587	2.683	8.951	4099	0.666	2.972	8.959	3865	0.533	2.832
18	Isoquercetin	9.228	4574	0.647	2.679	9.182	4668	0.550	2.693	9.189	4696	0.550	2.797
19	K-3-O-βR	10.437	6280	0.597	2.337	10.399	6003	0.373	2.285	10.402	6071	0.361	2.317
20	Naringin	11.009	8451	1.786	3.553	10.978	8729	1.897	4.113	10.978	8811	1.975	4.261
21	Hesperidin	11.495	8761	1.077	3.313	11.466	9162	1.266	3.886	11.465	9140	1.284	3.776
22	Daidzein	12.358	13358	0.395	2.126	12.332	14063	0.499	2.215	12.333	13816	0.404	2.205
23	Trans-cinnamic acid	12.749	45275	0.255	1.950	12.719	47365	0.384	2.075	12.720	47448	0.295	2.104
24	Quercetin dihydrate	12.999	7423	0.359	2.056	12.977	8034	1.629	2.095	12.977	8022	0.957	2.215
25	Naringenin	13.925	13065	0.316	2.167	13.905	13839	0.434	2.314	13.905	13835	0.336	2.357
26	Apigenin	14.041	17240	0.130	1.699	14.024	18187	0.195	1.726	14.024	18054	0.155	1.741
27	Kaempferol	14.143	10491	0.223	1.756	14.128	10972	0.280	1.784	14.128	10976	0.231	1.798
28	2-MC	14.358	32624	0.549	1.762	14.340	33852	0.404	1.807	14.340	33499	0.255	1.787

**Table 3:** System suitability parameters, correlation coefficients, LOD and LOQ of the method.

Sr. No.	Compound	System Suitability							Correlation Coefficient	Linearity Range (μg/mL)	LOD (μg/mL)	LOQ (μg/mL)
		RT	RSD	Area	RSD	USP Resolution	USP Tailing	USP Plate Count				
1	Gallic acid	0.739	0.2	11215	0.91		1.43	5384	0.998	2–6	0.080	0.264
2	3,5 DHBA	1.256	0.3	4009	1.44	11.25	1.46	10198	0.995	6–18	0.400	1.320
3	3,4 DHBA	1.460	0.2	7211	1.23	3.91	1.40	12274	0.996	2–6	0.150	0.495
4	Catechol	2.421	0.0	6245	1.04	15.42	1.27	19593	0.995	2–6	0.200	0.660
5	Chlorogenic acid	3.100	0.1	5562	0.37	8.97	1.08	24397	0.996	2–6	0.200	0.660
6	Catechin	3.232	0.1	3938	0.53	1.57	1.46	23646	0.997	4–12	0.450	1.485
7	Caffeine	3.589	0.2	15622	0.55	4.31	1.21	34002	0.997	2– 6	0.080	0.264
8	Syringic acid	4.246	0.2	14732	0.29	7.74	1.19	37086	0.998	2–6	0.100	0.330
9	Procyanidin B2	4.609	0.2	4456	2.26	3.63	1.26	29671	0.998	2- 6	0.300	0.990
10	Vanillin	5.186	0.1	23682	0.31	5.39	1.11	41111	0.997	2–6	0.200	0.660
11	Trans-*p*-coumaric acid	5.634	0.1	21509	0.31	4.25	1.15	46333	0.997	2–6	0.080	0.264
12	Salicylic acid	6.605	0.1	5213	1.67	7.62	1.41	32991	0.994	4–12	0.400	1.320
13	Trans-ferulic Acid	7.028	0.1	16178	0.46	3.23	1.10	63206	0.997	2–6	0.150	0.495
14	Trans-sinapic acid	7.501	0.1	7192	0.18	4.25	1.08	76938	0.996	2–6	0.400	1.320
15	Taxifolin	7.918	0.1	11366	0.44	3.59	1.08	69063	0.996	2- 6	0.080	0.264
16	Ellagic acid	8.130	0.1	8676	2.07	1.72	1.63	74926	0.995	2–6	0.400	1.320
17	Rutin hydrate	8.997	0.1	3836	1.22	7.80	1.09	133785	0.997	2–6	0.400	1.320
18	Isoquercetin	9.228	0.1	4574	0.61	2.26	1.11	127943	0.996	2–6	0.300	0.990
19	K-3-O-βR	10.437	0.0	6280	3.71	12.98	1.15	269816	0.997	2- 6	0.300	0.990
20	Naringin	11.009	0.0	8451	0.62	6.76	1.05	258318	0.998	2- 6	0.150	0.495
21	Hesperidin	11.495	0.0	8761	0.17	5.59	1.09	297168	0.998	2- 6	0.150	0.495
22	Daidzein	12.358	0.0	13358	0.43	10.33	1.10	378827	0.997	2–6	0.150	0.495
23	Trans-cinnamic acid	12.749	0.0	45275	0.13	4.33	1.05	274170	0.997	2–6	0.080	0.264
24	Quercetin dehydrate	12.999	0.0	7423	0.28	2.79	1.24	430037	0.996	2–6	0.400	1.320
25	Naringenin	13.925	0.0	13065	0.24	12.73	1.07	764867	0.997	2–6	0.150	0.495
26	Apigenin	14.041	0.0	17240	0.27	1.90	1.13	990979	0.994	2–6	0.150	0.495
27	Kaempferol	14.143	0.0	10491	0.21	1.81	1.18	1050030	0.996	2–6	0.200	0.660
28	2-MC	14.358	0.0	32624	0.47	3.66	1.05	899325	0.997	2–6	0.040	0.132

**Table 4:** Spiked amount and recoveries in sample C and D at three concentration levels.

Sr. No.	Compound	Spiked Amount (μg/mL)			Recovery % (Sample C)			Recovery % (Sample D)		
		75%	100%	125%	75%	100%	125%	75%	100%	125%
1	Gallic acid	3	4	5	102.06	96.28	95.53	100.83	100.54	93.47
2	3,5 DHBA	9	12	15	101.69	98.00	96.58	100.13	99.22	92.66
3	3,4 DHBA	3	4	5	105.81	101.53	99.91	103.01	102.39	96.35
4	Catechol	3	4	5	90.29	88.74	91.24	104.56	103.04	96.83
5	Chlorogenic acid	3	4	5	105.18	101.84	100.05	104.61	103.13	96.08
6	Catechin	6	8	10	106.97	103.34	101.20	103.43	103.61	96.21
7	Caffeine	3	4	5	94.11	93.09	93.62	111.86	103.63	96.72
8	Syringic acid	3	4	5	105.63	101.81	100.67	103.43	103.10	96.18
9	Procyanidin B2	3	4	5	103.99	101.05	99.12	101.57	102.74	93.68
10	Vanillin	3	4	5	92.92	92.19	92.63	90.54	93.03	88.13
11	Trans-*p*-coumaric acid	3	4	5	93.46	93.29	93.86	104.07	103.29	96.98
12	Salicylic acid	6	8	10	108.88	101.88	102.82	101.01	102.64	98.79
13	Trans-ferulic acid	3	4	5	93.38	92.61	93.79	90.88	93.90	88.86
14	Trans-sinapic acid	3	4	5	105.12	101.19	100.08	103.27	103.19	95.87
15	Taxifolin	3	4	5	105.45	101.96	100.57	103.73	102.86	96.12
16	Ellagic acid	3	4	5	104.55	100.46	100.02	102.01	101.39	94.23
17	Rutin hydrate	3	4	5	106.91	103.03	102.01	100.60	102.81	96.45
18	Isoquercetin	3	4	5	105.70	101.14	100.28	101.86	101.86	95.25
19	K-3-O-βR	3	4	5	84.85	85.41	86.52	86.59	86.81	84.95
20	Naringin	3	4	5	105.43	101.18	100.35	102.77	102.83	95.58
21	Hesperidin	3	4	5	94.34	92.91	93.21	104.43	103.58	96.13
22	Daidzein	3	4	5	92.03	91.27	92.08	89.38	92.19	87.70
23	Trans-cinnamic acid	3	4	5	105.93	102.18	100.70	103.32	103.03	95.92
24	Quercetin dihydrate	3	4	5	88.30	88.62	90.79	84.89	89.65	84.86
25	Naringenin	3	4	5	91.58	91.32	92.10	88.73	92.23	87.64
26	Apigenin	3	4	5	108.33	104.63	103.17	104.91	104.71	98.04
27	Kaempferol	3	4	5	105.96	102.63	101.07	103.24	103.18	96.52
28	2-MC	3	4	5	90.95	91.14	92.41	88.17	91.69	87.12

**Table 5:** Precision of the method.

Sr. No.	Compound	Precision				Intermediate Method Precision			
		Spiked Sample C		Spiked Sample D		Spiked Sample C		Spiked Sample D	
		RT (%RSD)	AREA (%RSD)	RT (%RSD)	AREA (%RSD)	RT (%RSD)	AREA (%RSD)	RT (%RSD)	AREA (%RSD)
1	Gallic acid	0.4	0.58	0.1	3.19	0.3	2.05	0.3	4.0
2	3,5 DHBA	0.3	0.46	0.0	1.12	0.3	0.75	0.4	2.38
3	3,4 DHBA	0.3	0.74	0.0	1.25	0.3	0.82	0.4	1.09
4	Catechol	0.2	0.68	0.1	0.83	0.2	1.05	0.3	1.15
5	Chlorogenic acid	0.2	0.75	0.2	0.95	0.4	1.33	0.5	1.84
6	Catechin	0.2	0.73	0.2	1.62	0.4	1.01	0.5	2.09
7	Caffeine	0.2	0.12	0.2	1.20	0.4	0.56	0.5	1.26
8	Syringic acid	0.2	0.51	0.1	0.62	0.4	0.49	0.4	1.04
9	Procyanidin B2	0.2	2.96	0.2	1.99	0.4	2.42	0.4	2.07
10	Vanillin	0.1	0.14	0.1	1.34	0.3	0.5	0.3	1.27
11	Trans-*p*-coumaric acid	0.1	0.69	0.1	0.81	0.3	0.58	0.3	1.34
12	Salicylic acid	0.1	1.89	0.1	4.09	0.2	1.58	0.3	3.06
13	Trans-ferulic acid	0.1	0.57	0.1	0.97	0.2	0.40	0.2	1.22
14	Trans-sinapic acid	0.1	0.89	0.1	0.75	0.2	1.35	0.3	1.97
15	Taxifolin	0.1	0.38	0.1	1.25	0.2	0.33	0.3	1.34
16	Ellagic acid	0.1	1.35	0.1	2.21	0.2	1.31	0.3	1.94
17	Rutin hydrate	0.1	2.38	0.1	2.34	0.2	1.63	0.3	2.33
18	Isoquercetin	0.1	0.91	0.0	1.95	0.2	1.10	0.3	1.91
19	K-3-O-βR	0.1	1.14	0.0	1.11	0.2	0.93	0.2	1.19
20	Naringin	0.1	0.35	0.0	0.80	0.1	0.74	0.1	1.96
21	Hesperidin	0.1	0.54	0.0	1.20	0.1	0.49	0.1	1.25
22	Daidzein	0.0	0.26	0.0	1.44	0.1	1.10	0.1	1.62
23	Trans-cinnamic acid	0.0	0.17	0.0	1.04	0.1	0.32	0.1	1.19
24	Quercetin dehydrate	0.0	0.89	0.0	1.47	0.1	0.96	0.1	1.94
25	Naringenin	0.0	0.23	0.0	1.29	0.1	0.67	0.0	1.43
26	Apigenin	0.0	0.15	0.0	1.16	0.0	0.46	0.0	1.33
27	Kaempferol	0.0	0.21	0.0	0.92	0.0	0.36	0.0	1.04
28	2-MC	0.0	0.81	0.0	1.76	0.0	0.93	0.0	1.46

## Supplementary Materials

The following are available online at https://www.mdpi.com/2218-273X/10/1/6/s1, The figures **A(1–3)** Chromatograms of peak purity: 1- Peak purities of standards, 2- Peak purities of spiked sample C, 3- Peak purities of spiked sample D,**B (1–28)** Linear curves obtained through Empower 3 software at 278nm: 1- Gallic acid, 2- 3,5 Dihydroxy benzoic acid, 3-3,4 Dihydroxybenzoic acid, 4-Catechol, 5-Chlorogenic acid, 6-Catechin, 7-Caffeine, 8-Syringic acid, 9-Procyanidin B2, 10-Vanillin, 11- Trans-coumaric acid, 12-Salicylic acid, 13-Trans-ferulic acid, 14-Trans-sinapic acid, 15-Taxifolin, 16-Ellagic acid, 17-Rutin hydrate, 18-Isoquercetin, 19-Kaempferol-3-O-βrutinoside, 20-Naringin, 21-Hesperdin, 22-Daidzein, 23-Trans-cinnamic acid, 24-Quercetin dihydrate, 25-Naringenin, 26-Apigenin, 27-Kaempferol, 28-2-Methoxycinnmaldehyde and **C (1–2).** Chromatogram of LOD (C.1), LOQ (C.2) obtained at 278nm.
