# Supplementary material for: Simultaneous Estimation of Twenty Eight Phenolic Compounds by a Novel and Expeditious Method Developed on Quaternary Ultra-Performance Liquid Chromatography System with a Photodiode Array Detector
Source: Biomolecules. 2019 Dec 18;10(1):6. doi: 10.3390/biom10010006 (PMC7023218; doi:10.3390/biom10010006)
Supplement: Supplementary file 1 [file biomolecules-10-00006-s001.pdf]

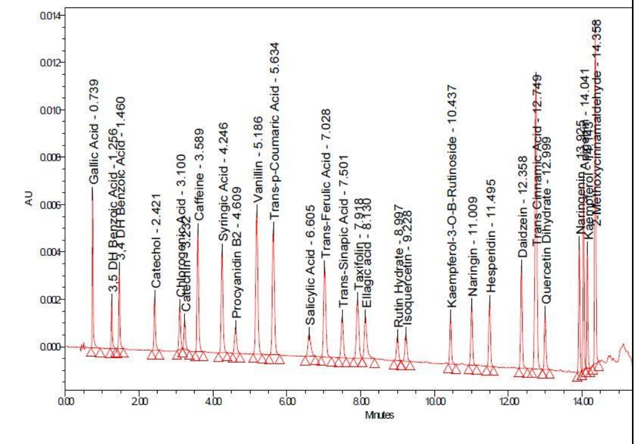

A. 1 Peak purities of standards

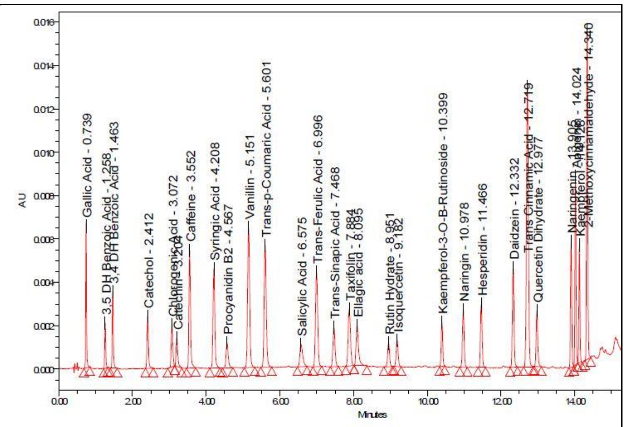

A. 2 Peak purities of spiked sample C

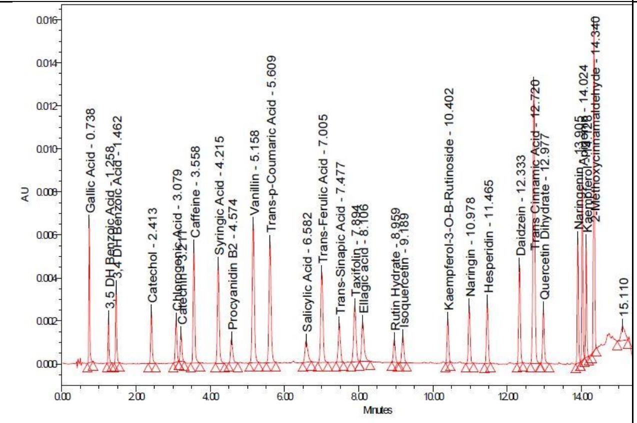

A. 3 Peak purities of spiked sample D

Figure A (1-3). Chromatograms of peak purity: 1-Standards peak purity, 2-Sample C spiked peak purity, 3-Sample D spiked peak purity

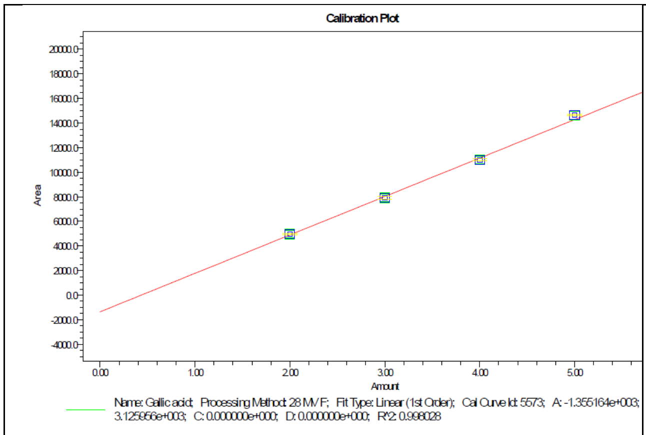

B. 1

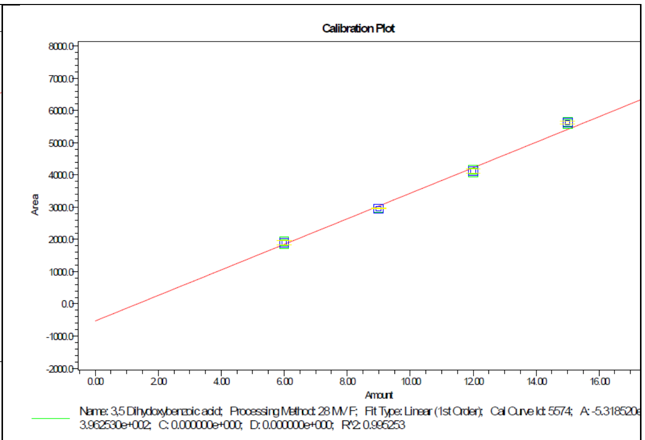

B. 2

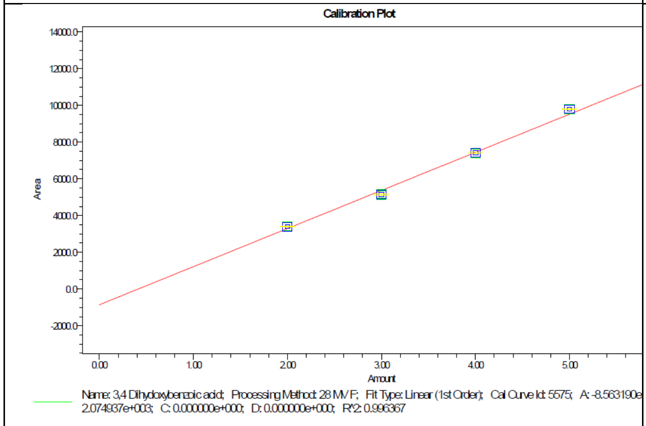

B. 3

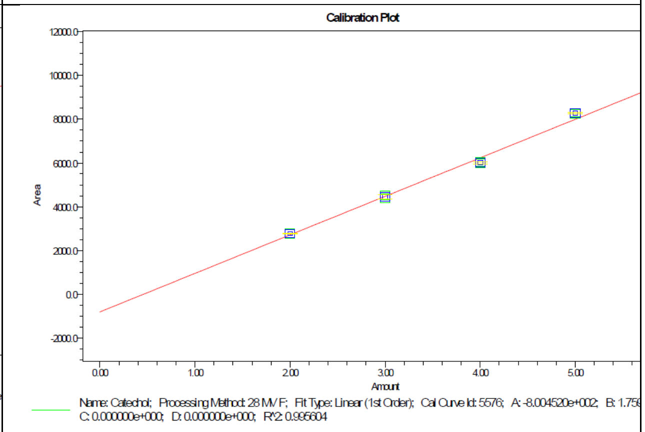

B. 4

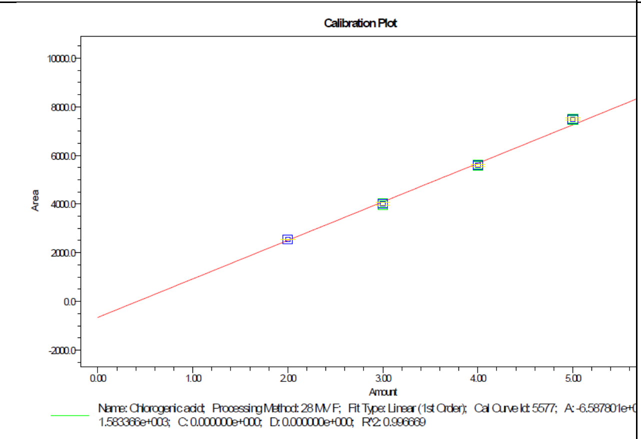

B. 5

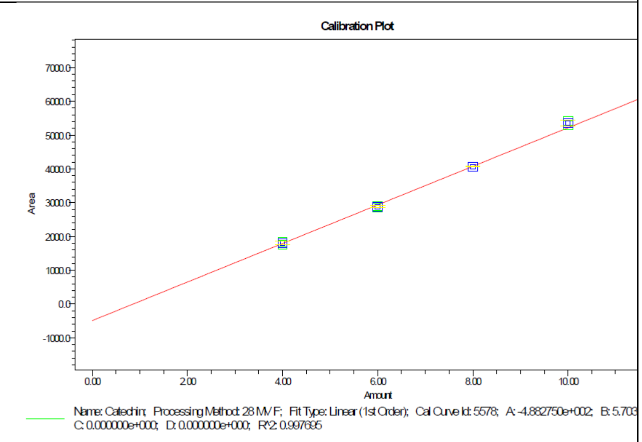

B. 6

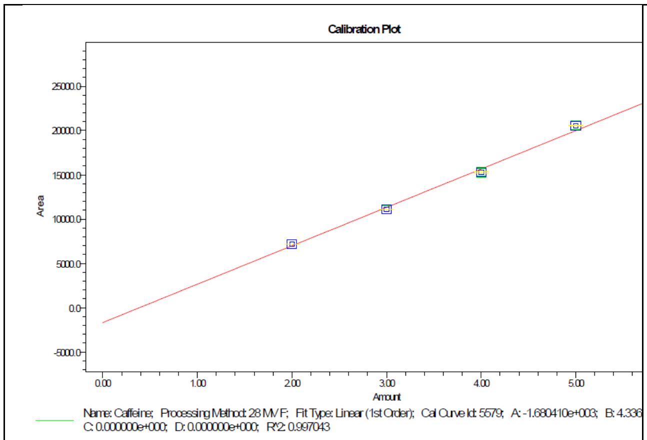

B. 7

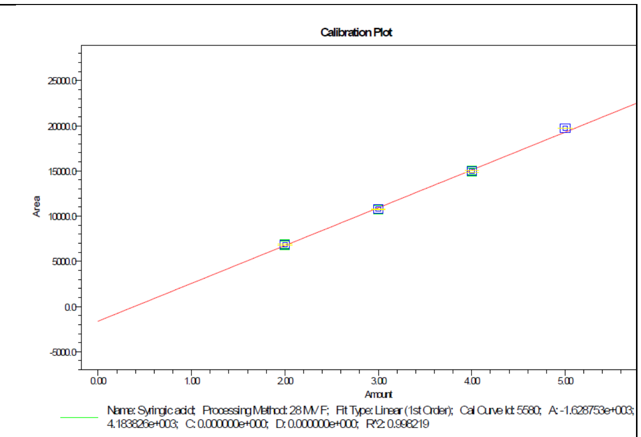

B. 8

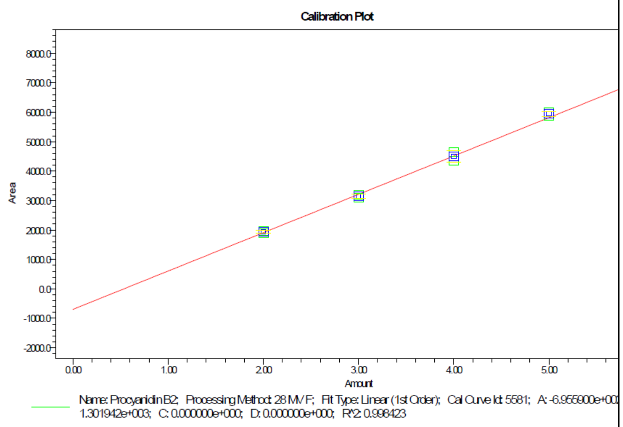

B. 9

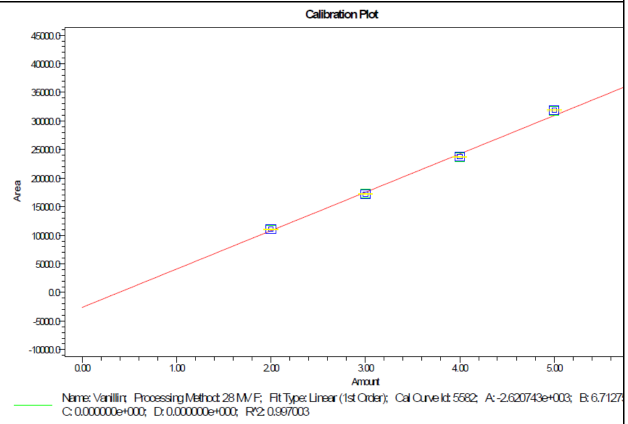

B. 10

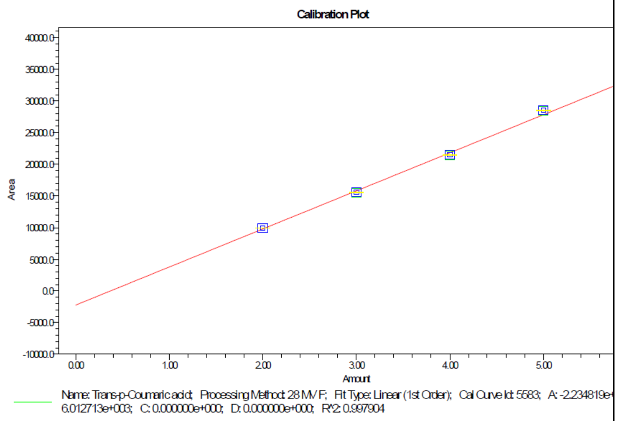

B. 11

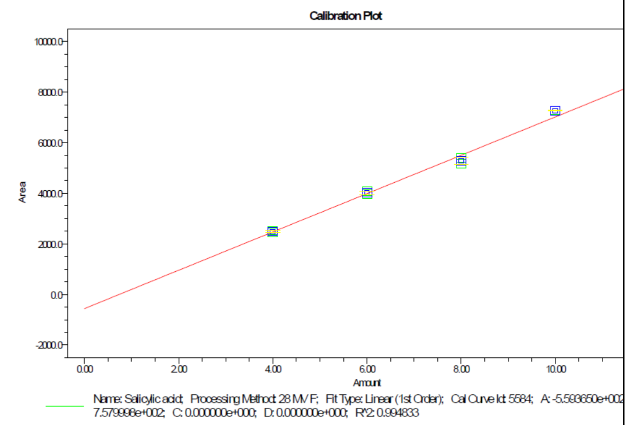

B. 12

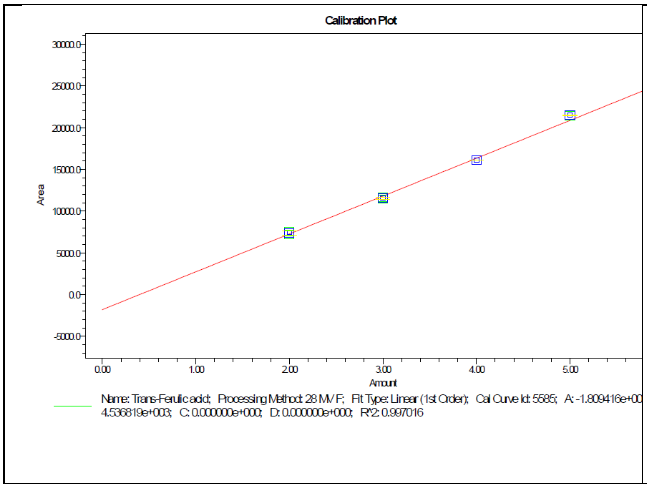

B. 13

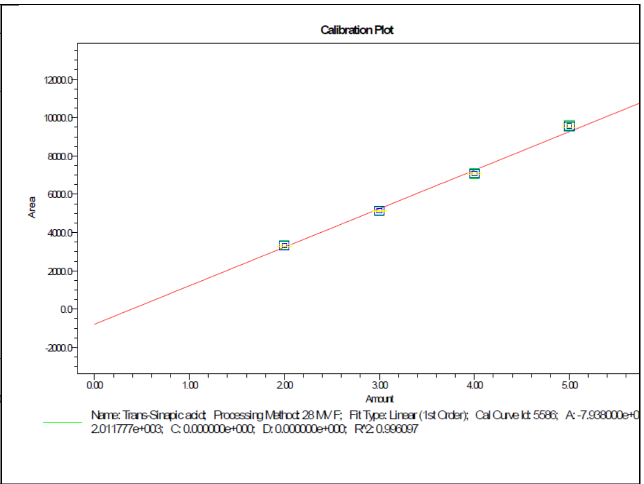

B. 14

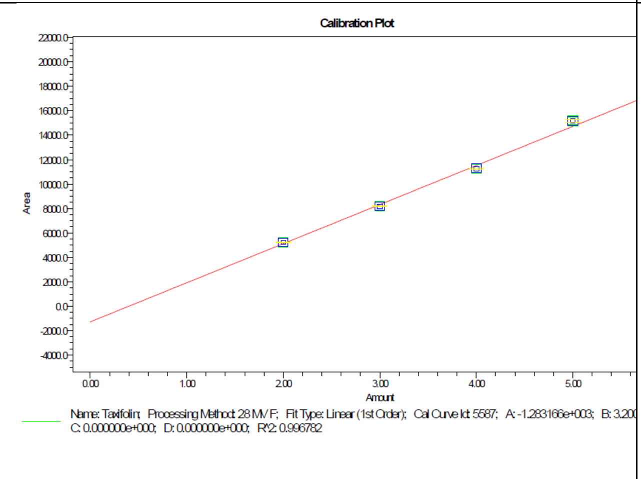

B. 15

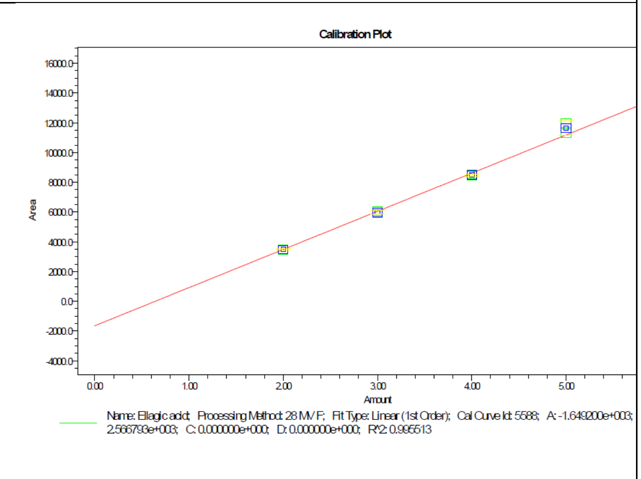

B. 16

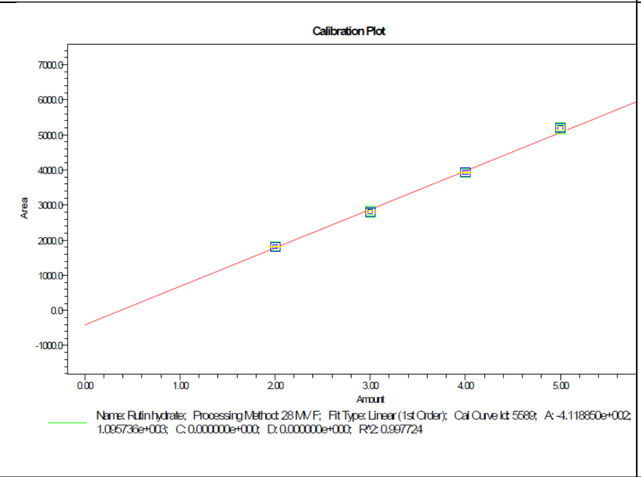

B. 17

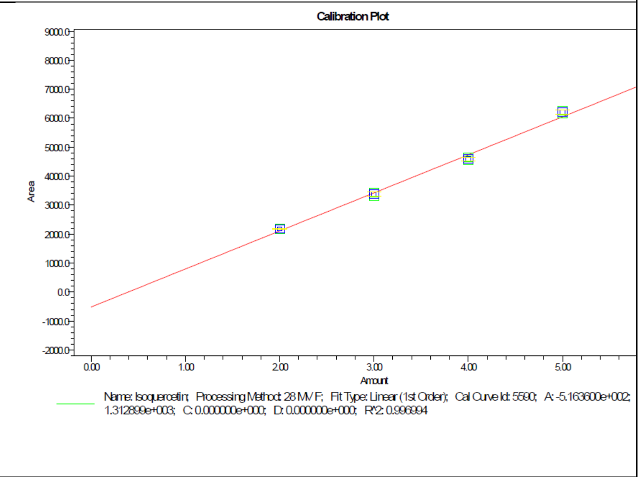

B. 18

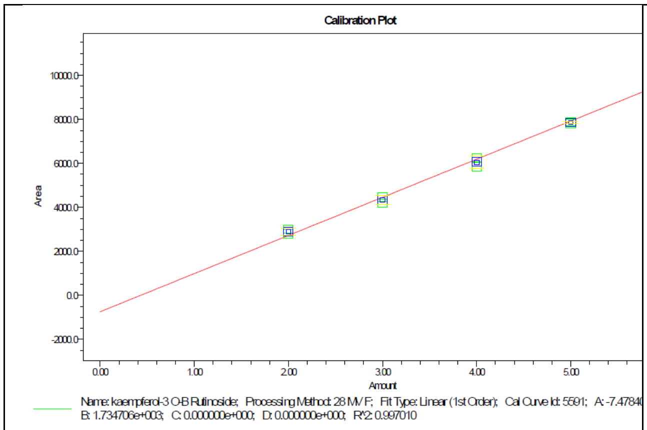

B. 19

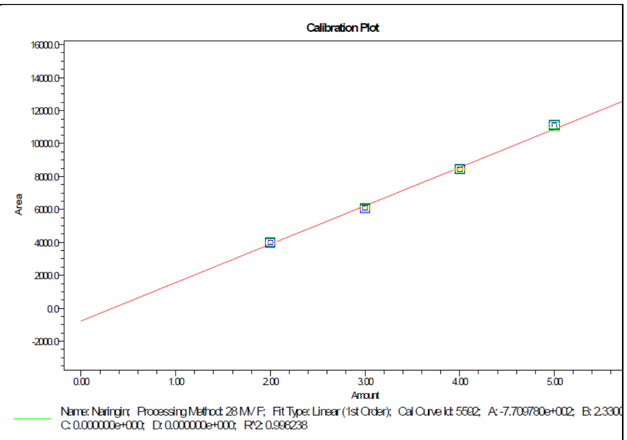

B. 20

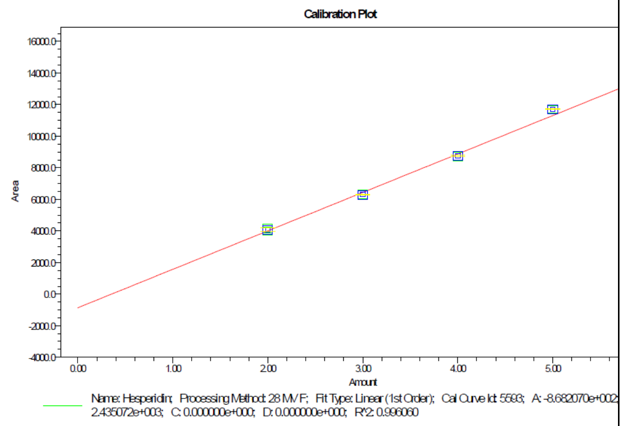

B. 21

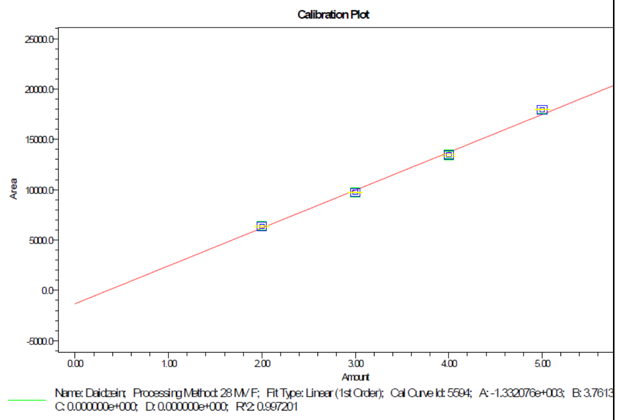

B. 22

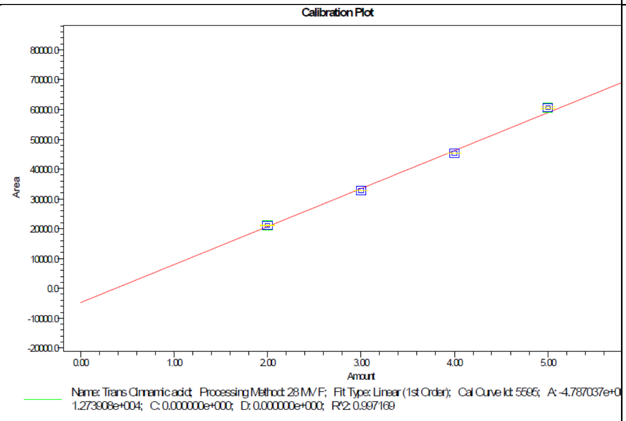

B. 23

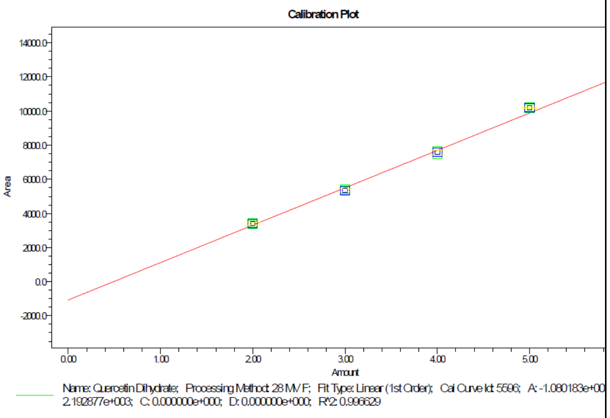

B. 24

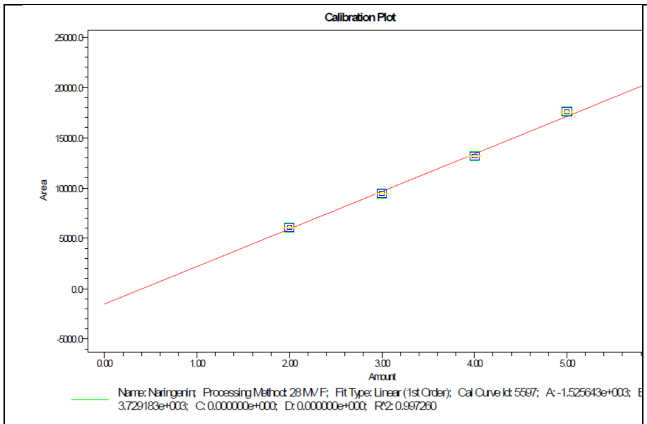

B. 25

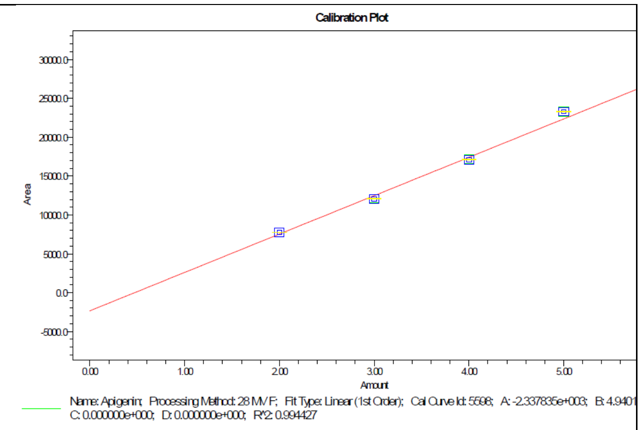

B. 26

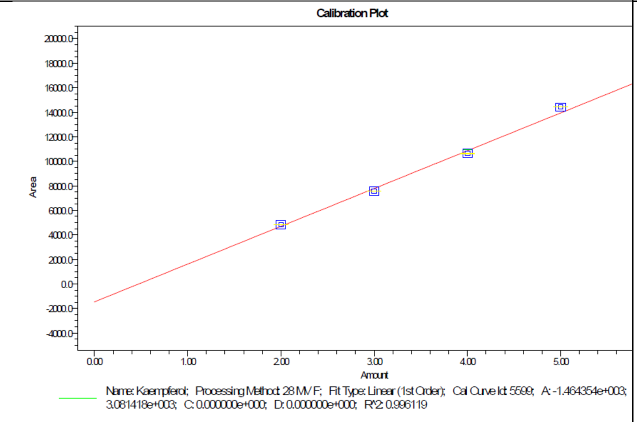

B. 27

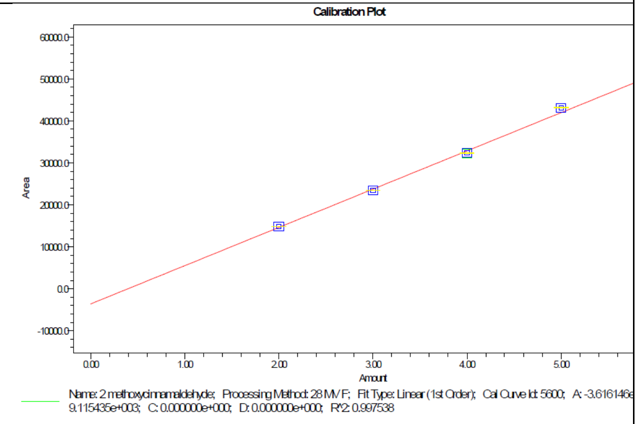

B. 28

Figure B (1-28). Linear curves obtained through Empower 3 software at 278nm: 1- Gallic acid, 2- 3,5 Dihydroxy benzoic acid, 3-3,4 Dihydroxy benzoic acid, 4-Catechol, 5-Chlorogenic acid, 6-Catechin, 7-Caffeine, 8-Syringic acid, 9-Procyanidin B2, 10-Vanillin, 11- Trans-coumaric acid, 12-Salicylic acid, 13-Trans-ferulic acid, 14-Trans-sinapic acid, 15-Taxifolin, 16-Ellagic acid, 17-Rutin hydrate, 18-Isoquercetin, 19-Kaempferol- 3-O-β rutinoside, 20-Naringin, 21-Hesperdin, 22-Daidzein, 23-Trans-cinnamic acid, 24-Quercetin dihydrate, 25-Naringenin, 26-Apigenin, 27-Kaempferol, 28-2-Methoxycinnmaldehyde

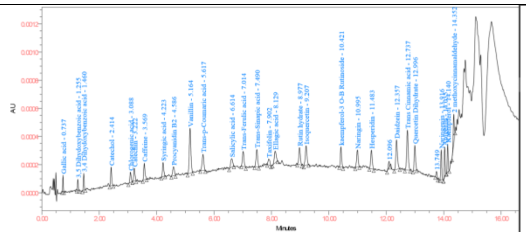	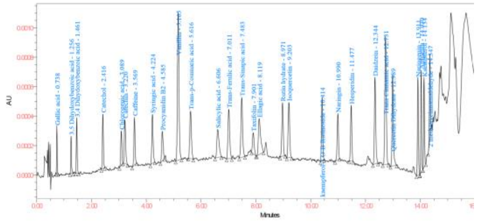
C. 1 LOD	C. 2 LOQ

Figure C (1-2). Chromatograms of LOD (C.1) and LOQ (C.2) obtained at 278nm.
